# Causes and Consequences of Ordering and Dynamic Phases of Confined Vortex Rows in Superconducting Nanostripes

**DOI:** 10.3390/nano12224043

**Published:** 2022-11-17

**Authors:** Benjamin McNaughton, Nicola Pinto, Andrea Perali, Milorad V. Milošević

**Affiliations:** 1 School of Science and Technology, Physics Division, University of Camerino, 62032 Camerino, Italy; 2 Department of Physics, University of Antwerp, Groenenborgerlaan 171, B-2020 Antwerp, Belgium; 3 Advanced Materials Metrology and Life Science Division, INRiM (Istituto Nazionale di Ricerca Metrologica), Strade delle Cacce 91, 10135 Turin, Italy; 4 School of Pharmacy, Physics Unit, University of Camerino, 62032 Camerino, Italy

**Keywords:** superconducting, nanostripes, vortex, confinement, critical current, flux

## Abstract

Understanding the behaviour of vortices under nanoscale confinement in superconducting circuits is important for the development of superconducting electronics and quantum technologies. Using numerical simulations based on the Ginzburg–Landau theory for non-homogeneous superconductivity in the presence of magnetic fields, we detail how lateral confinement organises vortices in a long superconducting nanostripe, presenting a phase diagram of vortex configurations as a function of the stripe width and magnetic field. We discuss why the average vortex density is reduced and reveal that confinement influences vortex dynamics in the dissipative regime under sourced electrical current, mapping out transitions between asynchronous and synchronous vortex rows crossing the nanostripe as the current is varied. Synchronous crossings are of particular interest, since they cause single-mode modulations in the voltage drop along the stripe in a high (typically GHz to THz) frequency range.

## 1. Introduction

Superconducting nanostripes (SNs) are a fundamental component in superconducting electronics, and they are crucial for various applications in the field of quantum technology. Superconducting nanostripe single-photon detectors (SNSPDs), for instance, are used for quantum communication and applications in astronomy and spectroscopy [1,2,3,4]. Other superconducting electronics include prototypical logic devices [5,6,7], flux qubits used in quantum computers [8,9,10], diodes [11,12,13], and electromagnetic resonators [14,15,16]. Narrow SNs experience an enhancement of critical parameters [17,18,19,20] due to confinement forces acting on the superconducting condensate [21,22,23,24,25,26]. Such confinement in narrow SNs can cause large magnetoresistance oscillations [27,28,29], where the time-averaged voltage/resistance, as a function of the applied magnetic field, exhibits pronounced peaks at alternating transitions between static and dynamic vortex phases. At higher applied fields, with multiple rows of vortices or high currents, a continuous motion of vortices causes a monotonic background on which the resistance oscillations due to entries of additional vortices are superimposed [27,30]. Commensurate effects between the SN width *w* and the number of vortex rows *n* are observed in the critical current as a function of the out-of-plane magnetic field *H* (for fixed *w*) or *w* (for fixed *H*) [31,32]. Optimised operation of some of the suggested superconducting electronics may be achieved on a specific geometry of vortices. For example, a single row of vortices was found to be preferable in [7], producing a giant non-local electrical resistance from vortices moving very far (several microns) from the local current drive. This effect appears to be important for a feasible long-range information transfer by vortices that are unaltered by the passing current. Moving vortices, however, exceed the bare transfer of information in importance. For example, vortices coherently crossing SNs can produce electromagnetic radiation [15,16], where higher radiation power is emitted when multiple vortices exit the SN simultaneously. In narrow SNs, rows of vortices can cross the SN asynchronously and synchronously [27,33], depending on competing forces (confinement, vortex–vortex interaction, Lorentzian forces), but the criteria for synchronous crossings are not yet well understood. In this respect, a study on the behaviour of vortices in SNs with small widths is important in order to reveal the favoured geometry of vortices for the static case (no sourced current) and the relation to the dynamic case (with sourced current). Understanding how a vortex lattice is affected by the interaction with the edge confining force and other dynamic forces is important when considering SNs for the applications mentioned above. Studying the dynamic dissipative states under strong confinement in the 1D–2D crossover regime can reveal how vortices cross a SN under different conditions (*w*, *H*, current intensity). Moreover, information on the possible vortex velocity under confinement [34,35] can be important for both fast information transfer and the frequency of radiation emitted by moving vortices.

In this work, we investigate how confinement in SNs affects the vortex configurations by using Ginzburg–Landau simulations [36]. We present a vortex row phase diagram as a function of *H* for a given *w*. An investigation of the dependence on the magnetic field of the average number of vortices reveals strong confinement effects. With increasing width, reconfiguration from the vortex rows to the vortex lattice takes place, offering a criterion for defining quasi-1D-to-2D dimensional crossover, where the SN effectively becomes a nanofilm in terms of its superconducting properties. Additionally, a commensurate behaviour of the critical current, $J_{c1}\left(H\right)$, was found when varying *H* by using a time-dependent GL approach in order to simulate the effects of the sourced current. We show that the values of the local minima in $J_{c1}\left(H\right)$ (defined as the onset of vortex motion and corresponding dissipation) are directly related to the row transitions shown in our vortex row phase diagram.

Further simulations of the current–voltage (I-V) characteristics in SNs evidenced transitions among different resistive regimes (Meissner, flux–flow, flux–flow instability, phase slips, normal state). I-V curves showing similar features to those of our simulations for SNs have been experimentally measured only for wider structures [37,38]. We find that for a SN with an average vortex density ≲$1/80\xi^{2}$ (with ξ being the coherence length) in a flux–flow regime, vortices cross the SN in a periodic/continuous fashion, causing modulations in the voltage drop that are experimentally detectable. Such a periodic flow may produce electromagnetic radiation [15] and features characteristic power spectra [39], which we report by performing fast Fourier transformation of the calculated voltage drop as a function of time during vortex motion. The recorded average vortex velocity (up to 10s of km/s) was used to discuss the washboard frequencies [15,16] in the flux–flow regime for thin SNs of niobium [38].

Provided that the vortex density is sufficiently high, as the sourced current density is increased, we show transitions of vortex row crossings from quasi-synchronous to synchronous. Synchronised crossings are desirable for small-band electromagnetic emitters operating in the GHz or THz range. For typical ultra-thin niobium SNs [18], the range of modulation frequencies in the microwave regime was between 10 and 800 GHz. Asynchronous regimes are disruptive for coherent emissions, but host a number of local dynamic vortical transitions and transformations that are of fundamental importance for advanced devices and are unattainable otherwise. The article is organised as follows. We first introduce the theoretical framework and methods used for the numerical simulations. We then present the results and discussions of all of the above-listed phenomena by using both stationary and time-dependent Ginzburg–Landau approaches. The main conclusions of our work are already emphasised in the section that provides the results before being additionally commented on in the conclusions of the article.

## 2. Materials and Methods

**Figure 1:**
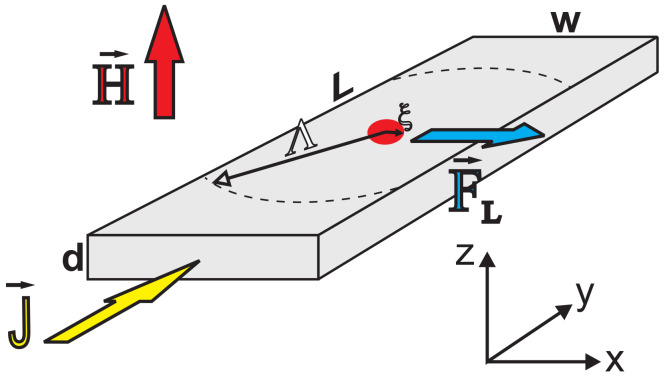
Schematic illustration of a superconducting nanostripe with width *w*, length *L*, and thickness *d* along the *x*, *y*, and *z* directions, respectively, in a homogeneous out-of-plane applied magnetic field, H. The SN contains an example of a single vortex, with a normal core of radius ∼ξ, and a distribution of magnetic field around it characterised by Λ. When a current density J is sourced, the vortex will experience the Lorentz force, $F_{L}$.

The numerical simulations performed in this work were all conducted on SNs such as that exemplified in Figure 1. The SNs have dimensions with a length *L*, width *w*, and thickness d≪ξ,λ, where ξ is the coherence length and λ is the magnetic field penetration depth of the superconducting state. For sufficiently large values of *H*, vortices form in the sample with a normal core of radius ξ and penetration of the magnetic field up to a characteristic length of λ. In the samples of our interest, which are very thin, the effective penetration depth $\Lambda =\lambda^{2}/d$ by far exceeds the dimensions of the SN, such that the magnetic response of the superconductor is negligibly small compared to the applied magnetic field. Simulations of such SNs were performed using the stationary (SGL) and time-dependent Ginzburg–Landau (TDGL) formalism. In the SGL approach, we self-consistently solve the coupled equations

(1) $${\left(-i\nabla -A\right)}^{2}\Psi =\Psi \left(1-{\left|\Psi \right|}^{2}\right),$$

(2) $$\vec{j}=-\kappa^{2}\nabla^{2}A=\frac{1}{2i}\left(\Psi^{*}\nabla \Psi -\Psi \nabla \Psi^{*}\right)-{\left|\Psi \right|}^{2}A$$

where Ψ is the superconducting order parameter, A is the vector potential, and κ=Λ/ξ is the effective Ginzburg–Landau parameter. We work with dimensionless units, where the length is given in units of the temperature-dependent coherence length ξ(T)=ξ, the vector potential A is given in units of cℏ/2eξ, the magnetic field $\vec{H}$ is given in units of the bulk upper critical field $H_{c2}=c\hbar /2e\xi^{2}$, the current is given in units of the GL current $j_{GL}=c\Phi_{0}/\left(8\pi^{2}\lambda^{2}\xi \right)$, and the order parameter Ψ is normalised to its value in the absence of an applied field or sourced current ($\Psi_{0}$). We impose the Neumann boundary condition at the superconductor–insulator boundary at the lateral edges of the SN:

(3) $$\vec{n}\cdot \left(-i\nabla -A\right){\Psi |}_{boundary}=0.$$

Along the length of the SN (*x*-axis), we enforce periodic boundary conditions for A and Ψ (for the unit cell length 32ξ, which is sufficient to capture the physics of interest in this work), of the form [40]:

(4) $$A\left(x_{0}+L_{x}\right)=A\left(x\right)+\nabla \chi_{f}\left(x\right)$$

(5) $$\Psi \left(x_{0}+L_{x}\right)=\Psi \left(x\right)\exp\left[i\frac{2e}{\hbar c}\chi_{f}\left(x\right)\right],$$

where $\nabla \chi_{f}$ respects the gauge used for the magnetic field. Equations (1) and (2) are solved numerically on a discretised Cartesian grid according to [36] by iteratively using the finite-difference method and the link–variable approach [41] until convergence within a prespecified error is achieved. Then, the supercurrent is calculated from the value of the order parameter and the vector potential (nearly entirely provided by the external magnetic field). With this method, we obtain the vortex row configuration–transition diagram as a function of *H* and *w* of the SN.

The generalised time-dependent Ginzburg–Landau formalism [42,43] should instead be employed to properly study the dynamical properties of the superconducting condensate (with order parameter Ψ(r,t)) in the presence of an external magnetic field H (with vector potential A) and sourced current density J, given by

(6) $$\begin{array}{c} \tau_{GL}N\left(0\right)\frac{u}{\sqrt{1-{\left(\Gamma |\Psi |\right)}^{2}}}\left[\frac{\delta \Psi }{\delta t}+i\frac{e^{*}}{\hbar }\varphi \Psi +{\left(\frac{\Gamma }{\sqrt{2}}\right)}^{2}\frac{{\delta |\Psi |}^{2}}{\delta t}\Psi \right]\\ =-\left(a+{b|\Psi |}^{2}\right)\Psi +\frac{\hbar^{2}}{2m^{*}}{\left(\nabla -ie^{*}A\right)}^{*}\Psi , \end{array}$$

(7) $$\nabla^{2}\varphi =\nabla \left[Im\left\{\Psi^{*}\left(\nabla -iA\right)\Psi \right\}\right],$$

where $a=\frac{\alpha }{2m^{*}\gamma }$, $b=\frac{\beta }{4m^{*2}\gamma^{2}}$, and $\Gamma =\frac{2\tau_{i}}{\hbar \sqrt{2m^{*}\gamma }}$. The Ginzburg–Landau order parameter’s relaxation time is $\tau_{GL}$; N(0) is the density of states at the Fermi level; the parameter u=5.79 in conventional superconductors; $e^{*}$ is the effective charge; φ is the electrostatic potential; $\tau_{i}$ is the electron–phonon inelastic scattering time; α,β,γ are material parameters. Equation (6) is solved by being coupled with the equation for the electrostatic potential (Equation (7)) by using Neumann boundary conditions at all sample edges, except for the leads into which sourced current is injected, where Ψ=0 and ∇φ=±J. This theory is derived for dirty gapless superconductors, where Cooper-pair breaking occurs due to strong inelastic electron–phonon scattering, and the physical quantities Ψ and *A* must relax over a time scale much longer than $\tau_{i}$. The distance over which an electric field can penetrate into the superconductor and the length over which relaxation processes occur are given by the characteristic inelastic diffusion length $L_{i}=\sqrt{D\tau_{i}}$, where *D* is the diffusion parameter, which is proportional to the electronic mean free path. In cases where $L_{i}<<\xi$, our simulations require very fine grid spacing (reflected in a consequently smaller time step in the implicit Crank–Nicolson method used) to yield physically correct results. In general, superconducting materials at *T* close to the superconducting-to-normal transition temperature, $T_{c}$, satisfy the conditions for the slow temporal and spatial variations that are ideally required for the applicability of the GL formalism. In the TDGL formalism, distances are given in units of ξ(T)=ξ; time is given in units of $\tau_{GL}=\frac{\pi \hbar }{8k_{B}T_{c}\left(1-T/T_{c}\right)u}$; temperature is given in units of $T_{c}$; the order parameter Ψ is given in units of $\Delta \left(0\right)=4k_{B}T_{c}u^{1/2}{\left(1-T/T_{c}\right)}^{1/2}/\pi$; φ is given in units of $\varphi_{GL}=\hbar /e^{*}\tau_{GL}$; the vector potential A is scaled to $A_{0}=H_{c2}\xi$; the current density is scaled to $J_{0}=\sigma_{n}\varphi_{0}/\xi$. The simulations are performed irrespective of the temperature $T/T_{c}$, and all physical quantities are scaled and normalised by reference quantities at a given temperature. Note that even though the GL approach is formally valid close to $T_{c}$, experiments have shown the possibility of extending the GL predictions to a finite *T* range below $T_{c}$ (see, e.g., [18] and the references therein). Moreover, in the simulations, the heat generated by the Joule effect is lost on a time scale shorter than the inelastic scattering time, assuming that the heat transfer coefficient is large enough to allow a fast dissipation. The approach adopted is equivalent to the inclusion of a solution of the thermal balance equation [34]. Our findings are valid at any temperature, provided that the coherence length is known at a given temperature.

## 3. Results

In what follows, by using SGL and TDGL simulations, we study how confinement forces in narrow SNs affect the stationary vortex configurations and their dynamics under *H* and a sourced DC current density, *J*.

### 3.1. Equilibrium Vortex Configurations

**Figure 2:**
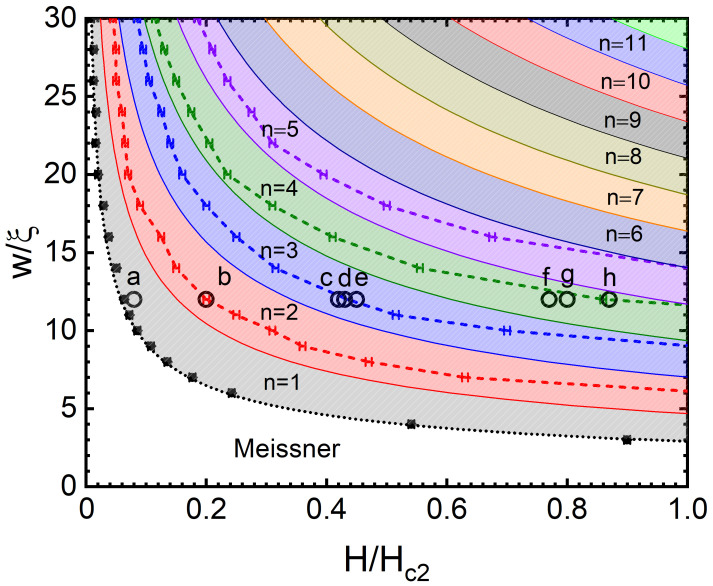
Equilibrium vortex row phase diagram that plots the SN’s width (in units of ξ) as a function of the intensity of the applied magnetic field (in units of $H_{c2})$ for different numbers of formed vortex rows (*n*). The simulations were performed using the SGL approach with periodic boundary conditions along the length, with a unit cell length of L=32ξ. The dashed lines denote the threshold for the formation of an additional vortex row (shown here up to n=5). The coloured regions represent the approximated regions for n>1, which are delimited by solid lines given by the expression $H_{row}/H_{c2}$ = $\frac{\pi n^{2}\xi^{2}}{\sqrt{3}w^{2}}$. The circles, which are labelled a–h, relate to the vortex configurations shown in Figure 3. The black dotted line corresponds to the analytical expression $H_{0}/H_{c2}=K\frac{\pi^{2}\xi^{2}}{2w^{2}}$ [44], with K=1.7 [45].

**Figure 3:**
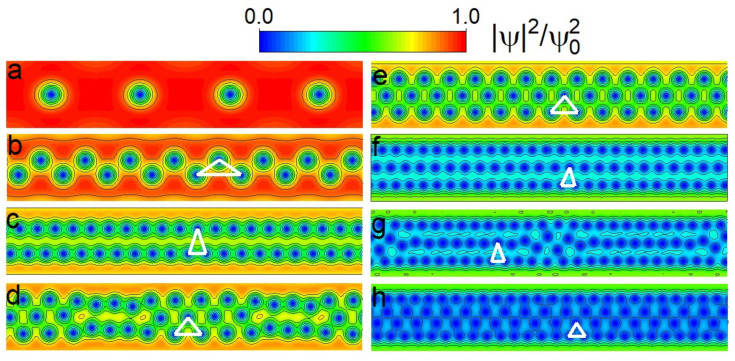
Calculated vortex configurations plotted as the Cooper-pair density for the ground state of a SN of width w=12ξ in a periodic cell of L=64ξ at different applied $H/H_{c2}$ values: (**a**) 0.08; (**b**) 0.20; (**c**) 0.42; (**d**) 0.43; (**e**) 0.45; (**f**) 0.77; (**g**) 0.80; (**h**) 0.87 (cf. Figure 2). Panels (**b**,**d**,**g**) depict the vortex states at the nucleation of a second, third and fourth row, respectively. Panels (**c**,**e**,**h**) show the most lattice-like packing conditions for two, three and four vortex rows, respectively. The white lines connecting the cores of three neighbouring vortices illustrate the deformation of the Abrikosov lattice [47] in the SN. The colour bar denotes the values of the Cooper-pair density shown in the panels. Each depicted configuration is indicated in Figure 2 with an open dot and is labelled accordingly.

We started by producing the vortex row phase diagram using the SGL approach, which showed the conditions for the formation of a number of vortex rows, *n*, as a function of *H* and *w* of the SN. Each dashed curve in the diagram shown in Figure 2, which plots the width of the SN versus *H*, represents the appearance of the $n^{th}$ vortex row (n=1÷5) in the ground state of the system as the magnetic field is increased. Examples of the corresponding vortex configurations for a SN of w=12ξ for different intensities of *H* are shown in Figure 3, and they correspond to the pinpointed dots (labelled a–h) in Figure 2.

To identify the threshold *H* for the transition to the vortex row configuration with a higher *n*, the ground states were first obtained for each SN at different values of *H*; then, the spatial distribution of the superconducting order parameter ${\left|\Psi \right|}^{2}$ was plotted (similarly to Figure 3) and carefully analysed, with a focus on the geometrical interpretation of the vortex configuration. In the SGL approach adopted in our simulations, the SN was considered periodic along its length, with a unit cell of L=32ξ. Several checks that were carried out by extending the unit cell length until 80ξ confirmed all of the following results.

Early theoretical works [44,46] showed that the magnetic field at which the surface barrier is suppressed and a single vortex can be stable in a SN is $H_{0}/H_{c2}=\pi^{2}\xi^{2}/2w^{2}$. The subsequent experimental observations of vortex penetration fields by Stan et al. [45] showed a very good agreement with that expression, up to a multiplying constant *K*. Our numerical data (black dots in Figure 2) reconfirm that finding, as the vortex penetration fields were found to nearly ideally match the same functional dependence on *w*, with a multiplying constant of K=1.7.

The approximate criteria for further reconfiguration of the vortex states and the appearance of additional vortex rows can be obtained in the following way. We consider an Abrikosov triangular lattice with the lattice parameter $a=1.075\sqrt{\phi_{0}/H}$. The vortices are arranged in a body-centred hexagonal lattice, so the Wigner–Seitz unit cell is hexagonal with a unit area per flux quantum of $A=\frac{\sqrt{3}}{2}a^{2}$. For a narrow SN, to accommodate *n* rows of vortices, the spacing, $w_{v}$, among the vortex rows must obey the inequality $w_{v}\leq w/n$. Using the previous expression for the Abrikosov vortex density, we substitute $A=\frac{\sqrt{3}}{2}w_{v}^{2}=\frac{\sqrt{3}w^{2}}{2n^{2}}$ to obtain the zeroth-order approximation for the threshold magnetic field required for the formation of new rows, yielding $H_{row}/H_{c2}$ = $\frac{\pi n^{2}\xi^{2}}{\sqrt{3}w^{2}}$. These approximate threshold *H* values are shown in Figure 2 by the solid lines that delimit different coloured regions, indicating transitions among states with different numbers of vortex rows. In general, the behaviour of threshold *H* found using the SGL simulations agrees well with the prediction of the formula. The values are, however, mostly higher than the approximate ones, which is attributed to the role played by the edge barriers for vortex entry and exit (varying depending on *w* and *H*). In addition, the rearrangement of the vortex lattice with every vortex penetration is not taken into account in the latter basic analytical formula. Note that such effects of the vortex–vortex interactions and interactions with the edge Meissner currents (causing the confinement force) dominate the formation of the vortex configurations in narrow SNs and present the main point of interest in this work.

For a SN of w=12ξ, we show different vortex row configurations in Figure 3 as they are formed in the ground state at different *H* values (marked by open dots in Figure 2). After the formation and growth of the population of the first vortex row (Figure 3a), *H* is increased, and the vortices are rearranged into a closely packed “zig-zag” state (Figure 3b). This close packing is emphasised by a white triangle that progressively deviates from the equilateral shape expected in the Abrikosov vortex lattice with the increase in *H*. Obviously, in this state, the Meissner currents will exert a strong repulsive and confining force on the vortices from the SN edges (i.e., a strong Bean–Livingston edge barrier [25]), resulting in a vortex spacing that is far smaller than the above rough analytical estimates (leading to the solid lines in Figure 2).

Starting from the one-row configuration (Figure 3a), further raising *H* and *n* strengthens the relevance of the vortex–vortex interaction forces for the resulting vortex configuration, which will increase the separation between the two rows (Figure 3c). At this point, we observe that additional vortices in the SN cannot uniformly balance the aforementioned competing force in the entire SN, leading to a local rearrangement of the vortex lattice to three rows (Figure 3d). Only by further increasing the field and having enough vortices in the SN can the full three-row state be formed (Figure 3e; notice the nearly ideal triangular lattice that is formed). For the considered width of the SN, the state with three vortex rows persists toward a much larger field due to quantum confinement, such that the vortices very strongly overlap in a closely packed structure (Figure 3f). Nevertheless, in the vicinity of the bulk upper critical field, a fourth row forms, first locally (Figure 3g) and eventually in the entire SN (Figure 3h), before the superconductivity is destroyed. No further rows of vortices can form with the higher field, and the existing vortex rows increasingly overlap until the normal state is established.

**Figure 4:**
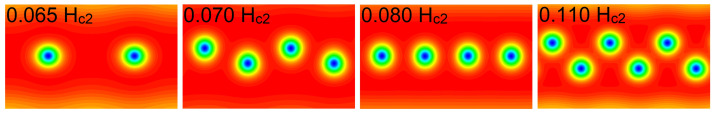
Example of a re-entrant transition between the one- and two-row vortex states in a SN of width w=20ξ caused by the competition between the confinement imposed by Meissner currents and the vortex density while both are changed with increasing *H* (values are indicated inside the panels).

We reiterate that the transitions among rows of vortices and the final arrangement of vortices in the lattice are strongly affected by the competition of the two forces, which are both dependent on *H*. As the magnetic field is increased, the edge Meissner current also increases up to the penetration of new vortices, while every new vortex changes the landscape of the vortex–vortex interactions in the SN. As exemplified in Figure 4 for w=20ξ, this nontrivial balance of competing forces can lead to a re-entrant behaviour in terms of the number of vortex rows formed. In such cases, the zig-zag instability of the vortex row can be “cured” back into a single row by increasing the Meissner currents, as the lateral confinement forces grow with the increase in the magnetic field. As *H* is increased further, the additional penetrating vortices tip the scale in favour of vortex interactions, and a definite reconfiguration into a state with two rows forms. This re-entrant behaviour was observed for nearly all SN widths considered in the range of w=20÷60ξ and only for the transition n=1→2. In such cases, we took the first onset of the zig-zag instability to mark the n=1→2 transition in Figure 2. Moreover, this range of widths in which such strong edge effects are detected marks the crossover from the quasi-1D to a 2D film-like behaviour.

**Figure 5:**
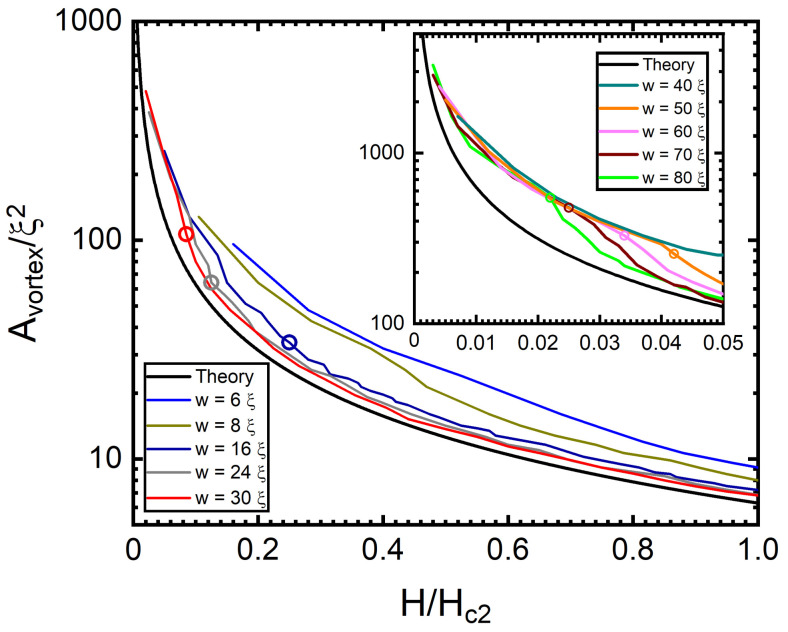
Area of the Wigner–Seitz unit cell containing a single vortex as a function of the applied magnetic field for a SN of width w=6÷30ξ and w=40÷80ξ in the inset, for small values of *H*. The analytical expression for the Abrikosov vortex lattice (AVL) area, $\frac{A}{\xi^{2}}$ = $2\pi \frac{H_{c2}}{H}$, is plotted as a black line. The open dots in each curve indicate the intensity of *H* for the formation of the third vortex row, above which the curves progressively approach the AVL expression when *w* is increased.

As the magnetic field is increased, vortices penetrate the SNs of different widths, vortex rows are formed, and a gradual evolution from a quasi-1D row pattern into a 2D vortex lattice is expected. To evaluate this crossover, we calculated the average area occupied by a single vortex as a function of *H* in all of the states found and compared this with the expected behaviour of the Abrikosov vortex lattice area. The strong confinement in the narrowest SN [25,31] dominates the vortex–vortex interaction, leading to the compression of the vortices into fewer vortex rows and, consequently, a larger average area per vortex. This can be seen in Figure 5 for w≤8ξ. As the width of the SN is made larger, the confining force from the edge current (at a given *H*) becomes less dominant with respect to the vortex–vortex interaction, resulting in a progressively closer agreement with the expected behaviour of a triangular vortex lattice [47]. This tendency is clearly visible upon the formation of the third vortex row (cf. Figure 5).

### 3.2. Vortex Dynamics under Sourced Current

All of the above results were obtained in the stationary case, where no current was sourced to the SN. A sourced current may change the stationary states or induce vortex dynamics that are specific to the nanoconfined regime. In what follows, we examine these non-equilibrium effects through TDGL simulations of time-dependent processes.

**Figure 6:**
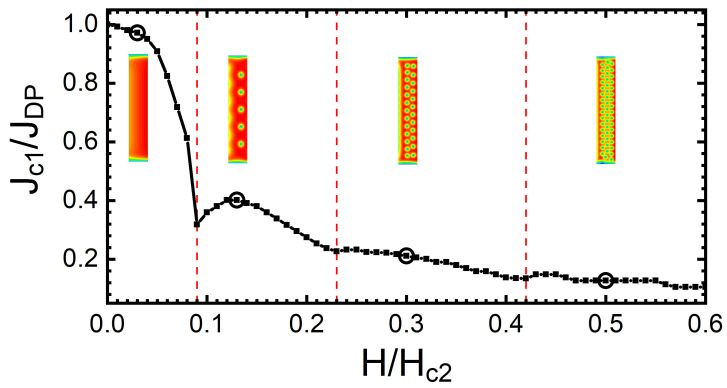
First critical current density normalised to $J_{DP}=0.385J_{GL}$ as a function of the applied magnetic field normalised to $H_{c2}$ for a SN of width w=12ξ, which was obtained by using the TDGL approach. Vertical red lines mark the transition to one-, two- and three-vortex-row states with magnetic fields of $H/H_{c2}$ = 0.09, 0.23 and 0.42, respectively. The insets illustrate the vortex row configurations with the selected magnetic fields (marked by open dots) for a sourced current density just below the critical one.

When a small transport current flows along a SN under an applied magnetic field $H\geq H_{c1}$, the present vortices experience a push across the SN, due to Lorentz-type force (∝J×H). As the current density is increased, vortices will continue to shift across the SN, finally leaving the stripe for a sufficiently large Lorentz force. This defines the first critical current density ($J_{c1}$) for which vortices are able to overcome the edge barrier [25,31] and start to cross the SN continuously, nucleating on one side, moving across the SN, and exiting at the opposing edge. The critical current depends on the magnetic field *H* for a given width *w* of the SN. The first critical current density as a function of *H*, $J_{c1}\left(H\right)$ is shown in Figure 6 for a SN of w=12ξ. A commensurate effect is observed between *H* and *n*, where the minima in the curve correspond to a transition to a state with an additional vortex row. Previous works have reported similar behaviours using different theoretical approaches [48,49], including a comprehensive study that used the TDGL approach [32] and revealed the relations between *n*, *w*, and the applied magnetic field [31]. Additionally, a similar effect related to this commensurability could be observed in the dependence on the magnetic field of the resistance, and this effect was reported in [7]. We included the study of commensurate effects in our work to emphasise the relation between the local minima and the transition to a new vortex row. The increase in $J_{c1}$ from the local minima as the applied field *H* is further increased is caused by the competition between vortex–vortex interactions and the confinement from the SN’s edge. After a local minimum, when a new row is formed, the vortex–vortex interactions are strong and the confining edge currents that produce an entry/exit barrier are reduced and are more easily overcome with lower sourced currents. As *H* is increased, the Meissner currents induced at the edge increase [50], reinforcing the edge barrier. The vortex row phase diagram in Figure 2 can be used to predict the transition field value, where local minima occur in the $J_{c1}\left(H\right)$ curves, which is an experimentally verifiable feature. Note, however, that the threshold fields for the formation of new rows in the presence of a sourced current are somewhat different from those presented in Figure 2, since the Lorentz push exerted by the current, effectively increases the confinement experienced by vortices prior to the onset of their motion.

**Figure 7:**
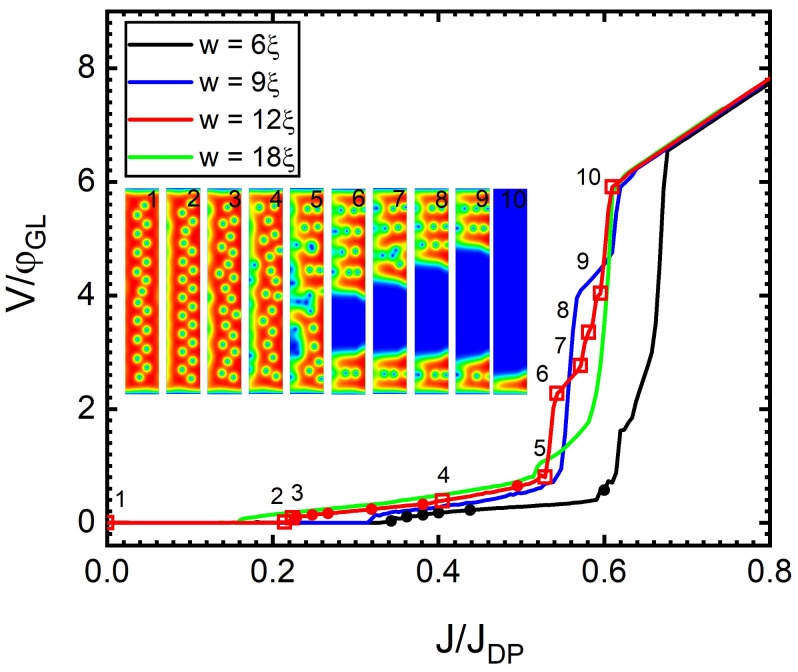
Normalised voltage drop as a function of the normalised current density for SNs of widths *w* = 6, 9, 12, and 18ξ with a magnetic field $H=0.25H_{c2}$. The black and red dots (for *w* = 6 and 12ξ, respectively) mark the values of the current density at which the analysis of the modulation frequency spectra are presented in Figure 9 and Figure 11. Inset: Snapshots of the Cooper-pair density for a SN of *w* = 12ξ, numbered 1–10 from left to right, are indicated by open red squares.

The TDGL approach also allowed us to simulate the voltage–current density (V-J) characteristics of SNs, which are presented in Figure 7 for stripes with *w* = 6, 9, 12, 18ξ, under an applied magnetic field $H=0.25H_{c2}$. The analysis of the V-J characteristics reveals a number of features related to different resistive regimes in each curve. At low values of *J*, stationary vortices are shifted to a new position across the SN due to the Lorentz force produced by the sourced current, so the resulting voltage drop and resistance remain zero. An example of this can be seen in Figure 7 (for w=12ξ) in the states labelled 1 and 2. When $J_{c1}$ is reached, the vortices cross the SN, and their perpetual motion leads to a finite resistivity value. Snapshots of this flux–flow regime can be seen in the states labelled 3 and 4 in Figure 7. By further increasing *J* and being in the presence of vortex–vortex interaction forces, a SN in the dissipative state exhibits flux–flow instability, where vortex cores interact during dynamics and the ordered lattice structure is lost during motion (the state labelled 5 in Figure 7). At even higher values of *J*, the vortices align during motion in a slip-streamed geometry (the vortices tailgate, i.e., subsequent vortices crossing the SN move in the wake of the previous vortex [33,35]) before a Langer–Ambegaokar phase slip [51] occurs across the SN. The normal area covered by the phase slip grows laterally with further increases in *J*, and additional steps in the V-J curve appear, with every slip-stream being merged with the growing phase slip, as seen in the states labelled 6–10 in Figure 7. When *J* reaches roughly 0.65$J_{DP}$, the SN transitions to a fully normal state with a linear ohmic behaviour. Similar V-I curves have been observed both numerically [52] and experimentally for Nb-C microstrips that were fabricated using focused-ion-beam-induced deposition [38]. In real materials, the presence of disorder and defects changes the behaviour described in this work. For example, edge defects are a favourable point for vortex entry, as current crowding occurs in the local defect region, which leads to favoured positions for vortex penetration. In addition, disorder on length scales larger than ξ may create bulk pinning regions [31]. On the other hand, small disordered regions (smaller than ξ) lead to increased inelastic scattering times, resulting in finite values of Γ. Hence, a viscous condensate will be formed, changing the dynamic behaviour of vortices and introducing additional resistive states, such as “vortex channels”. The appearance of such resistive states can be advantageous for EM emitters, provided that the vortex rows are synchronised, which we discuss next in the case of negligible disorder and defects.

**Figure 8:**
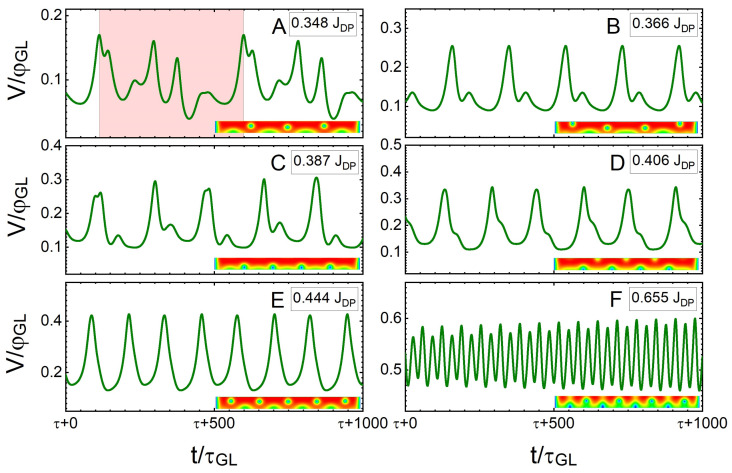
Normalised voltage drop as a function of time (normalised to $\tau_{GL}$) for a SN of w=6ξ under a magnetic field of $H=0.25H_{c2}$ sourced with different current densities of: (**A**) 0.348$J_{DP}$, (**B**) 0.366$J_{DP}$, (**C**) 0.387$J_{DP}$, (**D**) 0.406$J_{DP}$, (**E**) 0.444$J_{DP}$, and (**F**) 0.655$J_{DP}$. Each panel contains an illustrative snapshot of the spatial distribution of the Cooper-pair density during the dynamics. The pale red area in (**A**) shows the periodicity of the spectrum.

**Figure 9:**
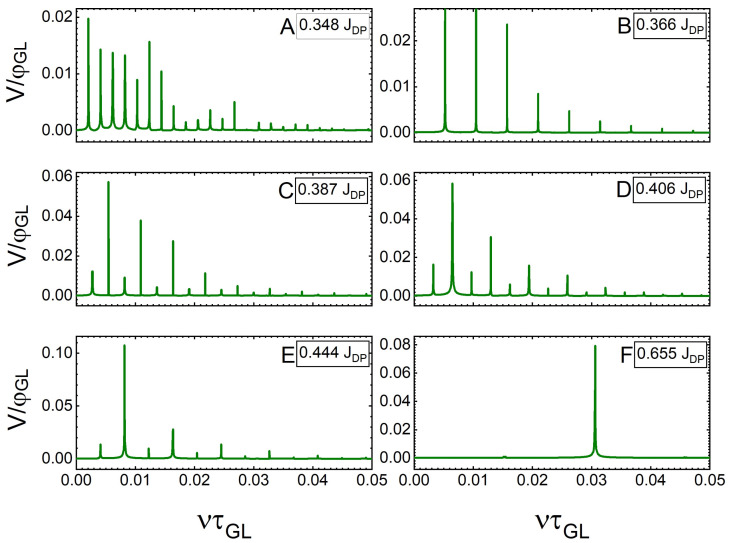
Spectra of the modulation frequencies ν (normalised to $\tau_{GL}^{-1}$) of the temporal voltage signals shown in Figure 8. (**A**) 0.348$J_{DP}$, (**B**) 0.366$J_{DP}$, (**C**) 0.387$J_{DP}$, (**D**) 0.406$J_{DP}$, (**E**) 0.444$J_{DP}$, and (**F**) 0.655$J_{DP}$.

**Figure 10:**
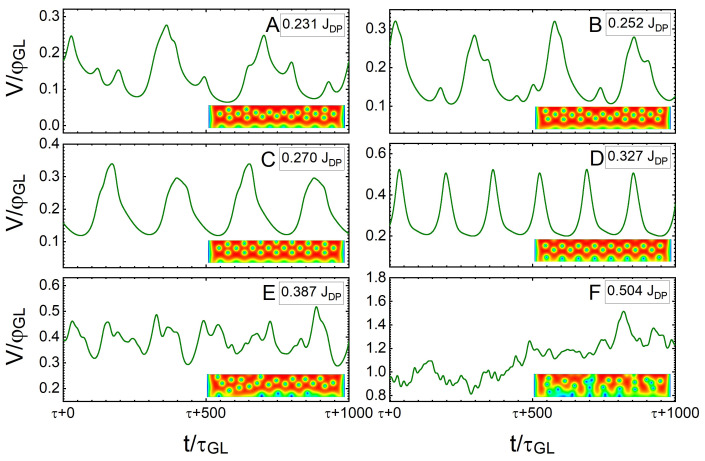
Normalised voltage drop as a function of time (normalised to $\tau_{GL}$) for a SN of w=12ξ under a magnetic field of $H=0.25H_{c2}$, sourced with different current densities: (**A**) 0.231$J_{DP}$, (**B**) 0.252$J_{DP}$, (**C**) 0.270$J_{DP}$, (**D**) 0.327$J_{DP}$, (**E**) 0.387$J_{DP}$ and (**F**) 0.504$J_{DP}$. Each panel contains an illustrative snapshot of the spatial distribution of the Cooper-pair density during the dynamics.

**Figure 11:**
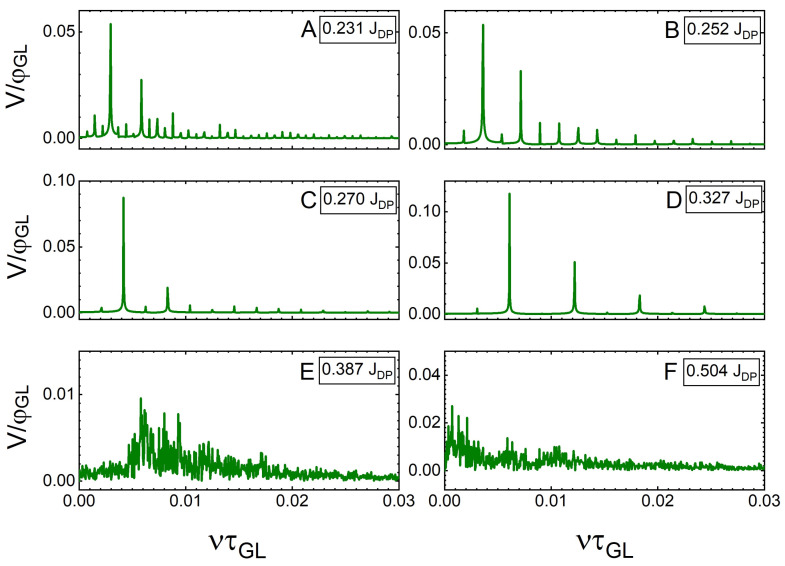
Spectra of the modulation frequencies ν (normalised to $\tau_{GL}^{-1}$) of the temporal voltage signals shown in Figure 10. (**A**) 0.231$J_{DP}$, (**B**) 0.252$J_{DP}$, (**C**) 0.270$J_{DP}$, (**D**) 0.327$J_{DP}$, (**E**) 0.387$J_{DP}$ and (**F**) 0.504$J_{DP}$.

Next, we discuss how the observed vortex crossings modulate the voltage drop across the SN and how synchronous and asynchronous crossings affect the spectrum of frequencies as a consequence of those modulations. In Figure 8, Figure 9, Figure 10 and Figure 11, we show the voltage as a function of time, V(t), for different sourced currents *J* and their corresponding spectra of frequencies (obtained by the Fourier transform of V(t)) for SNs of w=6ξ and w=12ξ under an applied field $H=0.25H_{c2}$. In each case, we first use the TDGL approach to find the ground states for each SN with the given magnetic field; then, we sweep *J* from 0 up to $≃J_{DP}$ in sufficiently small steps (typically $≃0.025J_{DP}$). At each current step, the simulation was left to run for a sufficiently long time such that a dynamic equilibrium was reached (typically up to $t=5\times 10^{3}$$\tau_{GL}$) before recording the data. The thus-obtained V(t) and the spatial distribution of the superconducting order parameter at each time step were used to produce Figure 8 and Figure 10.

In the dissipative state, V(t) increases as vortices move across the SN, with the maxima corresponding to the exit of a vortex and the minima corresponding to an entry of a vortex [34,53], leading to modulations of V(t) for both SNs considered (Figure 8 and Figure 10). Considering the SN of w=6ξ, which is sourced with the lowest current that causes the vortex crossing ($J=0.348J_{DP}$ in this case), V(t) shows evidence of asynchronous vortex dynamics, with several distinct features having a periodicity of 486 $\tau_{GL}$. Even though the vortices do not cross in synchronised rows, there is a quasi-synchronised behaviour that manifests in the repetition of vortex crossings in a given dynamic configuration. As *J* is increased from 0.348$J_{DP}$ to 0.406$J_{DP}$ (panels A–D in Figure 8), the modulations in the voltage evolve, and the number of modulations caused by quasi-synchronous crossings is reduced. Finally, beyond $J=0.444J_{DP}$ (panel E), there is only one mode that repeats periodically, i.e., the vortex dynamics become fully synchronous, and they accelerate with the further increase in the current (panel F). The relative spectra of the frequencies for V(t) are shown in Figure 9 in panels labelled correspondingly to the panels of Figure 8. The repetitive modes of vortex crossings within the particular dynamic configuration lead to peaks at specific frequencies. As the current density is increased, the spectra show an evolution to a single peak, corresponding to the frequency of $0.03\tau_{GL}^{-1}$.

A similar analysis for a SN with w=12ξ is shown in Figure 10 because a wider nanostripe allows the formation of multiple vortex rows in the ground state. Panel A of Figure 10, shows V(t) at $J=0.231J_{DP}$, when the vortices start dissipatively crossing the SN in a quasi-synchronous fashion. As *J* is increased to $0.327J_{DP}$, the vortex crossings become increasingly synchronised (panels B–D). However, at $J=0.387J_{DP}$, the flux–flow instability sets in (panel E) and causes an increasingly chaotic behaviour as *J* is increased to $J=0.504J_{DP}$ (panel F). In this regime, the apparent chaotic behaviour is caused by the competition between the standard vortex–vortex repulsion and the effective attractive core–core interaction due to preferential tailgating at large vortex velocities, which interchange their dominance over each vortex during the collective dynamics. For $J>0.52J_{DP}$, a phase slip occurs, which will grow as sourced current density is raised (shown in Figure 7 for the states labelled 6–10), and the remaining vortices cross the stripe in tailgated rows. This case of tailgated vortices causes periodic modulations V(τ); however, at such high values of *J*, this regime is unstable and, therefore, not considered in the following discussion. So, we only consider the region of strict flow–flow during the discussion of synchronised vortex crossings.

The spectra of the frequency modulations (Figure 11) show an analogous behaviour to that of the narrower SN discussed previously. At low values of *J*, when the crossings are quasi-synchronous, we see many mode contributions (i.e., few dominant peaks accompanied by many additional smaller peaks). As *J* is increased and synchronicity improves, the smaller contributions disappear, and the frequency component with the largest contribution is strengthened. However, at the onset of the flux–flow instability ($J=0.387J_{DP}$), we observe a broad contribution centred around the frequency $\nu =0.06\tau_{GL}^{-1}$ (corresponding to the median frequency of crossing of the vortex lattice as a whole, with many individual asynchronous crossings being superimposed). At $J=0.504J_{DP}$ (panel F), the spectrum loses all order, corresponding to the chaotic behaviour of vortex crossings.

Vortices that continuously cross the SN will cause oscillations in the electric and magnetic fields, leading to detectable emission of electromagnetic radiation [15,16,54]. The crossing of a single vortex releases a very small amount of energy, whereas multiple vortices moving coherently will emit a significant (and more easily detectable) amount of energy [54]. In a coherently moving lattice of vortices, periodic vortex crossing in the SN will cause the emission of radiation at a frequency of ω=2πv/a (washboard frequency) and at harmonics of ω=2πmv/a (m=2,3…), where *v* is the vortex speed and *a* is the lattice spacing (i.e., the distance between two parallel adjacent rows in our case) along the direction of motion [15]. The highest frequency emitted cannot exceed Δ/ℏ, where Δ is the superconducting gap of the SN. The theoretically predicted existence of radiation has been experimentally confirmed [16].

**Figure 12:**
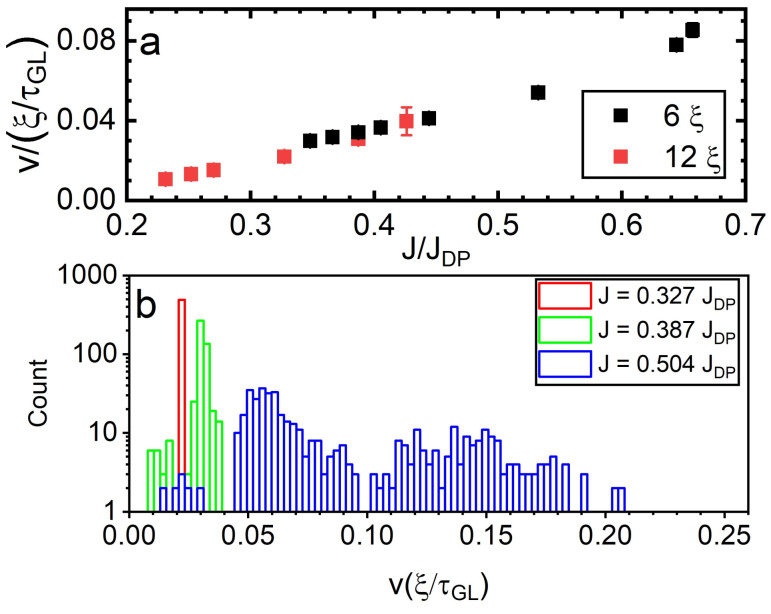
Panel (**a**)—Normalised average vortex velocity in units of $\xi /\tau_{GL}$ versus the normalised current density sourced to SNs of widths 6ξ and 12ξ under an applied magnetic field of $H=0.25H_{c2}$. Panel (**b**)—Histogram of vortex velocities for different values of *J* relating to different vortex crossing regimes.

The results of our simulations of the vortex velocity as a function of the sourced current density are shown in Figure 12a. They evidence a linear dependence at lower values of *J* for both of the narrow SNs considered above (which is similar to the behaviour seen in [52,55]). However, when *J* is increased to intermediate values, we find a deviation from the linear dependence, which is due to the increasingly facilitated vortex tailgating. We use these values of velocities and the frequency spectra to further discuss the potential for coherent radiation of vortices crossing the SN. In detail, considering the SN of width w=6ξ, at $J=0.348J_{DP}$, the average velocity is $v\approx 0.03\xi \tau_{GL}^{-1}$. The corresponding spectrum of modulations (Figure 9—panel A) shows a number of contributions, with the first five occurring at $\nu_{0}$ = 0.0021, $\nu_{1}$ = 0.0041, $\nu_{2}$ = 0.0062, $\nu_{3}$ = 0.0083, and $\nu_{4}$ = 0.0104 $\tau_{GL}^{-1}$, which are harmonics of the fundamental mode ($\nu_{0}$). The period of the cycle of repeating vortex crossings in this case is T = 486 $\tau_{GL}$, while the wavelength is 14.6ξ (obtained from λ=vt by using the value of the vortex velocity in Figure 12). As the vortices move in a quasi-synchronous manner, the washboard frequency [15] is not applicable.

As the sourced current is increased, the vortices cross the SN in a more synchronised manner. In this case, we can apply the relation for the washboard frequency to the values of the average vortex velocity *v* and the frequency of the first harmonic $\nu_{0}$ and obtain the value for the (virtual) lattice spacing *a*. By increasing *J* from $0.366J_{DP}$ to $0.655J_{DP}$, the values of this lattice spacing decrease from 6.2ξ to 2.8ξ, where the values are extrapolated from the washboard frequency relation. The combination of increasing Lorentz force and edge confining forces causes the reduction of *a* and an increase in the vortex density during the dynamics. However, in the wider SN (w=12ξ), we do not observe the same behaviour. In this case, the value of *a* remains constant (≃3.6ξ, obtained using the washboard frequency relation) as the current is increased in the dissipative state, until it transitions to asynchronous crossings for high values of *J*. At this point, the resultant force on the vortices no longer leads to a preferred geometrical dynamic configuration. A combination of the Lorentz force, edge Meissner current confining forces, vortex–vortex interactions, and vortex nucleation rate results in the asynchronous behaviour. This suggests that in the wider SN, the vortex–vortex repulsion within the lattice is more deterministic for the resulting lattice spacing *a* than the interactions with confining edges during the vortex dynamics.

The synchronisation of vortex crossing in a fixed lattice with large sourced currents was observed and discussed in [33], albeit without identifying an exact regime as such.

**Figure 13:**
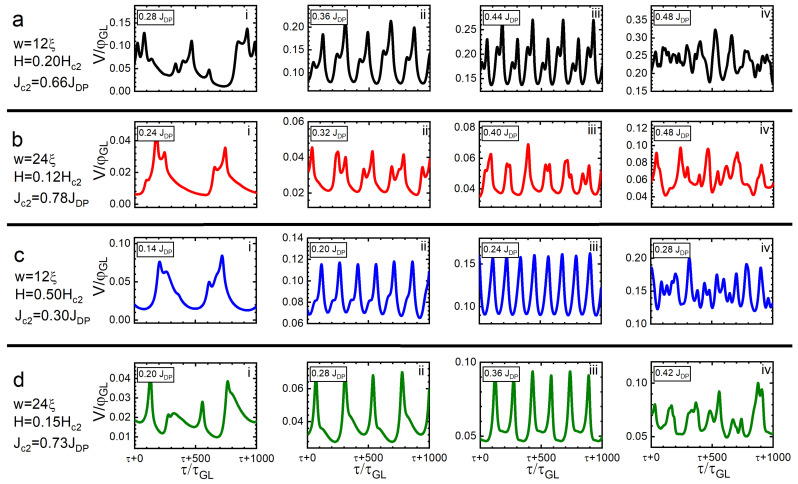
Temporal evolution of the normalised voltage drop with increasing (indicated) values of the sourced current density for two SNs of widths 12ξ and 24ξ. All plots exhibit the voltage modulations caused by vortex crossing. Panels (**a**). i–iv: w=12ξ, $H=0.20H_{c2}$ (single row of vortices). Panels (**b**). i–iv: w=24ξ, $H=0.12H_{c2}$ (two vortex rows). Panels (**c**). i–iv: w=12ξ, $H=0.50H_{c2}$ (three rows). Panels (**d**). i–iv: w=24ξ, $H=0.15H_{c2}$ (three rows). The first panel in each row corresponds to the onset of the dissipative state; the second and third belong to the synchronous/quasi-synchronous regime; the fourth is the onset of the flux–flow instability regime.

To better understand the regime in which synchronous lattice crossings can occur, we performed a number of additional simulations. Figure 13 shows four other examples of the modulations in V(t), corresponding to a SN with w=12ξ and $H=0.2H_{c2}$ (panels a.i–iv), which realises a single row of vortices in the static case (Figure 2). At $J=0.28J_{DP}$, the SN becomes dissipative with asynchronous vortex crossing behaviour (a.i), until the point at $J≃0.36J_{DP}$, where quasi-synchronous crossings begin (a.ii). The latter continues (a.iii) until $J=0.48J_{DP}$, where the flux–flow instability sets in (a.iv), achieving the normal state at $J_{c2}=0.66J_{DP}$. Panels b.i–iv of Figure 13 show similar behaviours for a SN of width w=24ξ in an applied magnetic field $H=0.12H_{c2}$ (realising two vortex rows), which does not transition to synchronised crossings as the sourced current is increased. However, after increasing the magnetic field applied to either SN, synchronous crossings will take place (panels c.i–iv and d.i–iv). For SNs of *w* = 12 and 24ξ at *H* = 0.50 and 0.15$H_{c2}$, respectively, the vortex configuration comprises three parallel rows. For w=12ξ (w=24ξ), synchronous crossings start at $J≃0.20J_{DP}$ ($J=0.28J_{DP}$) and continue until the onset of flux–flow instability at $J=0.28J_{DP}$ ($J=0.42J_{DP}$).

One can conclude that the frequency of the radiation stemming from coherent vortex crossings can be tuned with *H* and/or with *J*. The applied magnetic field *H* changes the vortex density (affecting the number of rows) and, hence, *a*, while the transport current directly changes the vortex velocity. Both factors influence the behaviour of vortex crossings, which, in turn, affect the electromagnetic radiation emitted at frequencies ν=v/a [33]. For an insight into the values expected in the experiment, we consider the parameters measured for Nb thin films by Pinto et al. [18]. For example, in a Nb film of thickness *d* = 20 nm, ξ(0)≃ 8.0 nm, and $T_{c}=8$ K, given a Ginzburg–Landau time of $\tau_{GL}≃65$ fs, our results show an average velocity of vortices crossing the Nb SN of thickness 20 nm and width 50–100 nm to be in the range 1–10 km/s, with the first harmonic frequencies being in the range of 1–50 GHz. These values are similar to those reported by Dobrovolskiy et al. [16,38] and to those of Embon et al. [35]. A thin and narrow superconductor with a high value of $T_{c}$ and a small value of $\tau_{GL}$ (≃1−10 fs), in which faster vortex crossings could be realised, could be used as a terahertz radiation source. Such sources are highly sought for a variety of applications [56], including clinical applications [57] and terahertz time-domain spectroscopy [58].

## 4. Conclusions

In summary, our comprehensive study details how lateral quantum confinement in superconducting nanostripes (SNs) leads to experimentally detectable physical effects in the spatial configurations and dynamical phases of vortices and vortex rows. In the stationary case, we presented an equilibrium vortex row phase diagram in the parametric space delimited by the width of the nanostripe and the applied magnetic field. The diagram showed how the narrowest SNs support a lower number of vortex rows up to relatively large values of the applied magnetic field, thus exhibiting a lower average vortex density (for given values of *H*) that deviates more from the theoretical value attributed to the vortex density in an Abrikosov lattice. Although the phase diagram predicts the number of rows in an SN under a given magnetic field without referring to specific superconducting materials, our work provides an indication of the range of microscopic parameters that can be found in several families of superconductors, which deserves to be exploited in order to design novel and advanced quantum devices. At intermediate SN widths, i.e., w=20÷60ξ, we pointed out that an interplay of vortex interactions, Meissner currents, and the related edge barrier can lead to a re-entrant behaviour of the vortex row states (1→2→1) as the field is increased, which may lead to large magnetoresistance oscillations, as discussed in [30]. In relation to transport and dynamics, we reported that the critical current (for the very onset of dissipation) as a function of the magnetic field will exhibit oscillations commensurate with the formation of every additional vortex row. With sourced current beyond the critical value, the vortices crossing the SN modulate the voltage drop across the SN, which is also linked to the emission of electromagnetic radiation. More vortices crossing in synchronicity causes more radiation power to be emitted for that crossing frequency. We showed that depending on its width and the applied magnetic field a SN, under a sourced current, enters the dissipating state, with the vortices crossing relatively slowly and quasi-synchronously at first. As the current intensity is increased, the crossings become increasingly synchronous, until flux–flow instability occurs and synchronicity is gradually lost. Not every example showed the evolution toward synchronised vortex crossings; however, a regime for the occurrence of the crossing of vortex rows in a fixed lattice was observed for states with an average vortex area of $A≲80\xi^{2}$. This is also related to effective confinement, as a large vortex density is required so that the edge confining forces can adequately act on the vortex rows and lock them into a dynamically synchronous lattice, with typical crossing frequencies in GHz-to-THz range, which is beneficial for electromagnetic radiation in the corresponding frequency bandwidth.
